# *Streptococcus pneumoniae* Infection in Patients with Asplenia: A Spanish Perspective over a 25-Year Period

**DOI:** 10.3390/antibiotics13010104

**Published:** 2024-01-21

**Authors:** Enrique Gea-Izquierdo, Gil Rodríguez-Caravaca, Ruth Gil-Prieto, Valentín Hernández-Barrera, Ángel Gil-de-Miguel

**Affiliations:** 1 Department of Medical Specialties and Public Health, Rey Juan Carlos University, 28922 Madrid, Spain; 2 María Zambrano Program, Rey Juan Carlos University, European Union, 28922 Madrid, Spain; 3 Department of Preventive Medicine, Hospital Universitario Fundación Alcorcón, Rey Juan Carlos University, 28922 Madrid, Spain; 4 CIBER of Respiratory Diseases (CIBERES), Instituto de Salud Carlos III, 28029 Madrid, Spain

**Keywords:** asplenia, pneumococcal disease, disease burden, hospitalization, Spain

## Abstract

Anatomical or functional asplenia constitutes a risk factor for *Streptococcus pneumoniae* (SP) infection, being more frequent in children and the elderly and in people with multiple comorbidities. We aimed to describe the impact of invasive pneumococcal disease (IPD) on the clinical features and outcomes of patients hospitalized for asplenia in Spain. Discharge reports from the Spanish Minimum Basic Data Set were used to retrospectively analyze hospital discharge data with a diagnosis of asplenia from 1997 to 2021. A total of 132,257 patients with asplenia (splenectomized/non-splenectomized) were identified from the Spanish database. Among the cases, 177 (37.5%) patients with splenectomy and 295 (62.5%) patients without splenectomy developed IPD. The clinical presentations (non-infection vs. infection) did not significantly differ between the two reference groups, except for patients with COPD, rheumatoid disease, AIDS, other neurological disorders, metastatic cancer, and drug abuse. The risk factors for IPD were also more frequently reported in patients without splenectomy (*p* < 0.001) and with comorbidities (*p* = 0.005). The study of patients with asplenia provides relevant information about the state of SP infection. This epidemiological tracking can serve to better understand the comorbidities that affect them, the risk factors for the disease, the prediction of antibiotic use, and vaccination in public health, among other factors.

## 1. Introduction

Invasive pneumococcal disease (IPD) and pneumococcal pneumonia represent serious health problems among adults, older people, and those with certain conditions and pathologies, among which immunocompromised and some immunocompetent patients stand out, which makes them more susceptible to infection and favors a picture of greater severity and worse evolution [1]. Patients with functional asplenia (hyposplenism) have a high risk of infection. The prevalence of this disease and the frequency of overwhelming post-splenectomy infection (OPSI) vary based on condition, and the latter is more frequent. Sickle cell disease (SCD) is accompanied with hyposplenism in almost 100% of cases. Additionally, the Advisory Committee on Immunization Practices (ACIP) recommends that patients with asplenia receive pneumococcal vaccinations in addition to routine vaccinations [2].

*Streptococcus pneumoniae* (SP) is a bacterium responsible for a significant burden of diseases in the adult population, manifesting as localized forms, IPD (meningitis, sepsis, and bacteremic pneumonia), and non-bacteremic pneumonia as the most frequent diseases [3]. In recent decades, SP is one of the pathogens that has acquired greater antibiotic resistance. Resistance to beta-lactams and macrolides is especially important [4]. The appearance of cases due to non-susceptible serotypes has great clinical relevance due to these cases’ higher lethality. When SP infection is suspected in a patient with asplenia, intravenous antibiotics should be immediately administrated with no delay, and even diagnostic studies are needed [5]. Nonetheless, the fatality rates are high despite the use of antibiotics (in overwhelming sepsis, the fatality rates are 40–50%). Antibiotic prophylaxis and immunization against *Haemophilus influenzae*, *Neisseria meningitidis*, and SP are critical adjuncts in patients with hyposplenism [6].

IPD has been notifiable to the Spanish National Epidemiological Surveillance Network since 2015. Epidemiological surveillance focuses on the invasive forms, which are more lethal, and it has an essential role in assessing the effect of the vaccine on the incidence of the disease and the consequences of changes in circulating serotypes. Thus, we sought to describe the impact of IPD on the clinical features and outcomes of patients hospitalized for asplenia. The objective of this study was to determine the burden of disease due to asplenia and analyze the related diseases in Spain.

## 2. Results

**Figure 1:**
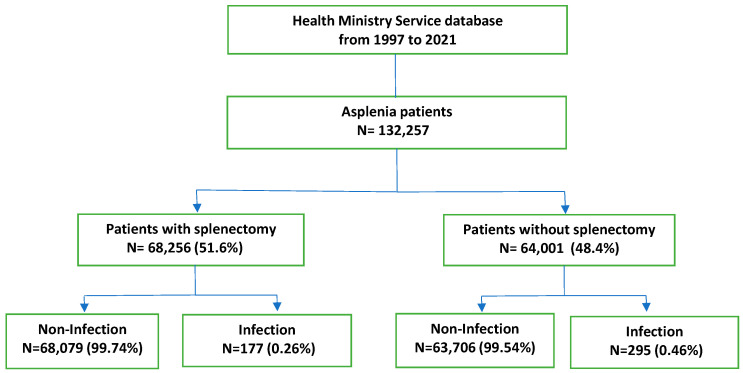
Flowchart of patients with asplenia.

From January 1997 to December 2021, 132,257 patients with asplenia were identified from the Spanish CMBD database. The flowchart of patients and IPD is shown in Figure 1.

The male-to-female ratio was 1.59, and the average age at the time of asplenia was 53.81 years. Among the cases, 177 (37.5%) patients with splenectomy and 295 (62.5%) patients without splenectomy developed IPD (472). For the latter, *Streptococcus pneumoniae* (481; J13) was the most common event (37.5% patients), followed by spleen trauma (865; D73), malignant neoplasm of the stomach (151; C16.9), and septicemia (38; A10, A11). In total, 72 (15.25%) IPD-related mortalities were found.

**Figure 2:**
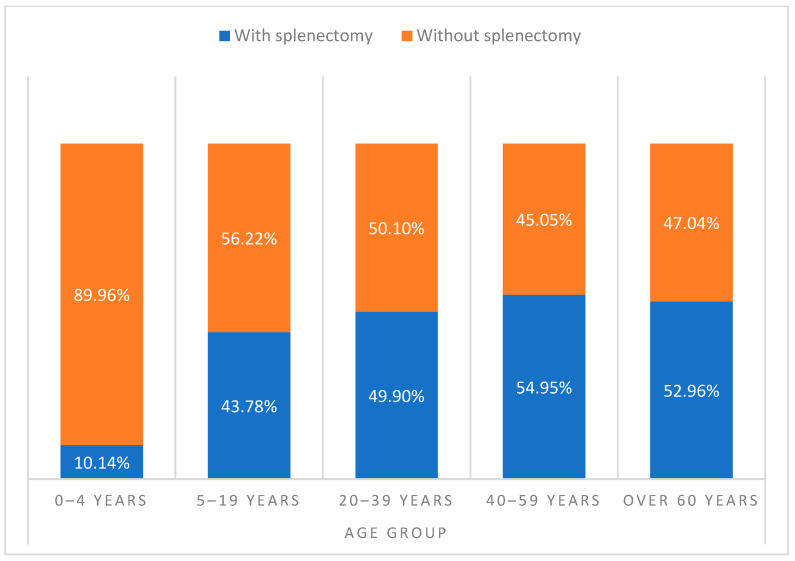
Distribution of the patients with asplenia by age group in Spain, from 1997 to 2021.

**Figure 3:**
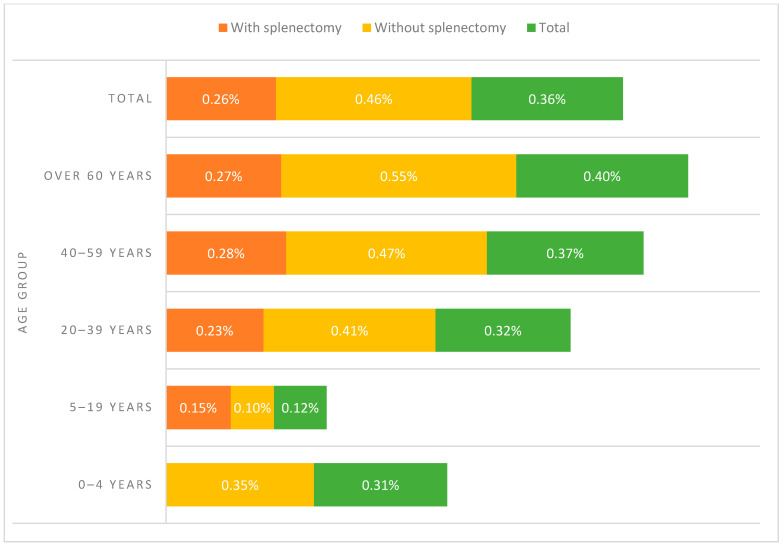
IPD prevalence of the patients with asplenia by age group in Spain, from 1997 to 2021.

Among the splenectomized patients who had an IPD episode, 164 (92.6%) had a total splenectomy and 13 (7.34%) a partial splenectomy. In the case of non-splenectomized patients who had an IPD, 45 (15.25%) showed congenital asplenia. The development of IPD differed between age groups when comparing patients aged 0–4, 20–39, and 40–59 years (*p* < 0.001, Figure 2). No relevant differences were identified regarding SP non-infection vs. infection and sex. The prevalence of pneumococcal disease was 1.67-times higher in non-splenectomized patients compared to splenectomized patients (N = 295/64,001, 0.46% vs. N = 177/68,256, 0.26%; *p* < 0.001) (Figure 3).

Clinical presentation did not significantly differ between the two reference groups (Table 1), except for COPD (non-infection, N = 11,858/11,938, 9% vs. infection N = 80/11,938, 16.95%; *p* < 0.001), rheumatoid disease (non-infection, N = 1671/1687, 1.27% vs. infection N = 19/1687, 3.39%; *p* < 0.001), AIDS (non-infection, N = 1206/1232, 0.92% vs. infection N = 26/1232, 5.51%; *p* < 0.001), other neurological disorders (non-infection, N = 3309/3333, 2.51% vs. infection N = 24/3333, 5.08%; *p* < 0.001), metastatic cancer (non-infection, N = 17,275/17,308, 13.11% vs. infection N = 33/17,308, 6.99%; *p* < 0.001), and drug abuse (non-infection, N = 2511/2537, 1.91% vs. infection N = 26/2537, 5.51%; *p* < 0.001) (Table 1). Table 2 and Table 3 provide a comparison of IPD patients who were splenectomized compared to those who were non-splenectomized. The risk factors for IPD were also more frequently reported in non-splenectomized patients (*p* < 0.001) and those with comorbidities (*p* = 0.005). Detailed data are provided in Table 4. Patients who were identified in the second group were more likely to have comorbidities (OR = 1.61 [95% CI: 1.2–2.16]) (*p* = 0.001) and the first to be male patients (OR = 1.63 [95% CI: 1.18–2.25]) (*p* = 0.003). Specially, the highest OR was in the age groups 40–59 and over 60 years. The most predominant comorbidities in patients suffering IPD as the first diagnosis were cancer (18.43%), COPD (16.95%), liver disease (13.56%), diabetes (10.59%), and alcohol abuse (10.17%). 

The annual number of claimed cases for asplenia tended to increase slightly over the years, with a significant trend (average annual percentage change = 0.2%; *p* < 0.001). In relation to comorbidities, patients had more than one (29.7%, N = 39,266), and the proportion of patients with any risk factor for IPD was 62.0% (N = 82,018).

LOHS (length of hospital stay) was higher in infected patients (18) for those who did not have IPD (11), accounting for 31% of the absolute difference in hospital stay (statistically significant; *p* < 0.001) (Table 1). In terms of patients with IPD, 36 (20.34%) of the splenectomized group died compared to 36 (12.20%) of the non-splenectomized patients.

## 3. Methods

### 3.1. Study Design

The population of interest was patients with diagnosed asplenic conditions. This nationwide database study examined patients with newly diagnosed asplenia. A 25-year national multicenter retrospective cohort study included patients admitted to Spanish hospitals for a pneumococcal infection. Patients with asplenia were analyzed according to the presence of SP infection.

### 3.2. Data Source and Study Population

An observational study was conducted to determine the Spanish burden of disease due to asplenia. We estimated the comorbidities associated with asplenia with special emphasis on IPD using a time-series period. Data were extracted from January 1997 to December 2021 from the Spanish population using the Spanish National Surveillance System for Hospital Data (CMBD). These data included all hospitalizations for 98% of Spanish hospitals [7,8], while cases were obtained through the Spanish public health surveillance system.

The Spanish Minimum Basic Data Set is a data model for hospital discharges, extending the registry to the private sector and other areas with hospitalization. Data were obtained from the Spanish Minimum Basic Data Set, which includes all territories (17 autonomous communities, including Ceuta and Melilla). We used discharge reports from the CMBD, published annually by the Spanish Ministry of Health (Government of Spain), to retrospectively analyze the hospital discharge data [7] starting from 1997. The data used for this research were certified by the Ministry of Health and statistically deidentified. The index date was the first date with a diagnosis or procedure code for asplenia during the identification period. The study population included adults aged 0 > 60 years who were diagnosed with immunocompromising conditions in the aforementioned period. At-risk individuals were divided into age strata, including 0–4, 5–19, 20–39, 40–59, and >60 years. Cases with asplenia, splenic dysfunction, or complement deficiencies were chosen.

### 3.3. Demographic and Hospitalization Data

Eligible patients had a diagnosis code for asplenia or a procedure code (Spanish Current Procedural Terminology (CPTSP)) for splenectomy (total or partial) during the patient identification period (Appendix A). 

Cases were categorized as splenectomy or non-splenectomy when patients with asplenia were revealed. Patients that were hospitalized for splenectomy were compared to patients with hyposplenic/congenital asplenia. The independent variable of interest was asplenia, and patients were categorized into those with or without a spleen intervention based on these data. Other variables studied were birthdate, diagnosis ranging from 1 to 14, Morphologies 1 and 2, intervention date, procedures ranging from 1 to 20, severity level, mortality level, age, readmission, cost, autonomous community, and nationality.

First-incidence hospitalizations with a diagnosis of asplenia were included in the study, avoiding double counting admissions. Concerning admission and during hospital stay, demographics data, laboratory findings, clinical reconnaissance, comorbidities, microbiological research, and therapeutic management information were collected. In splenectomized/non-splenectomized patients, the following data were obtained from the discharge reports: hyposplenia (SCD, splenic irradiation) or cause of asplenia (hematological diseases, cancer, or congenital, or postsplenectomy in case of trauma). The primary outcomes of interest were the occurrence of major hospital complications defined as comorbidities, the presence of myocardial infarction, congestive heart failure, peripheral vascular disease, cerebrovascular disease, dementia, chronic pulmonary disease, rheumatic disease, peptic ulcer disease, diabetes without chronic complication, diabetes with chronic complication, hemiplegia or paraplegia, renal disease, any malignancy including lymphoma and leukemia (except malignant neoplasm of skin), AIDS/HIV, pulmonary circulation disorders, hypertension (complicated), paralysis, other neurological disorders, liver disease, metastatic cancer, obesity, alcohol abuse, and drug abuse.

The clinical criteria for case definition of IPD was associated with cases produced by the systemic dissemination of SP. The clinical form of a patient´s disease presentation was not decisive in the case definition. Laboratory criteria included some of the following: isolation of SP in a normally sterile location, detection of SP nucleic acid in a normally sterile location, or detection of an SP antigen in a normally sterile location. Whenever possible, the strains should be serotyped [9]. Patients with pneumococcal infection (blood or cerebrospinal fluid cultures positive for SP, or positive pneumococcal urinary antigen testing) were included.

### 3.4. Statistical Analysis

Baseline variables were analyzed descriptively, and data were provided as numbers (%) and percentages for categorical variables and as means and standard deviations (SD) for continuous variables. Sociodemographic variables, clinical characteristics, comorbidities, risk factors, and IPD-related outcomes were compared between splenectomized and non-splenectomized patients using the Student’s *t*-test, Chi-square test, or Fisher’s exact test, as convenient. Logistic regression analysis was performed to identify the factors associated with infection risk after asplenia (age at diagnosis/surgery, sex, and comorbidities) and effect estimates (odd ratios). Analyses were adjusted for sex and age. The last was at the event, or at the last visit to the hospital or relatives in the case of no event.

In all tests, the significance level used was *p* < 0.001. Statistical analyses were performed using Stata Software (version 16.1.).

### 3.5. Ethical Statement

The study was conducted in accordance with the Declaration of Helsinki, principles of good clinical practice, and all applicable regulatory requirements. Informed consent and informed assent were not necessary. The patient information was anonymized and deidentified prior to the analysis. No formal ethics approval was required.

## 4. Discussion

The Spanish population studied showed a very balanced number of patients with and without splenectomy in both groups, and those infected with SP were <0.5%. The distribution of patients with asplenia presented great uniformity between splenectomized and non-splenectomized patients aged >5–19 years. Patients with asplenia suffering IPD have more severe infection than those with a functional spleen. However, no significant differences can be identified in mortality rate for patients with IPD and patients with a spleen. In patients with asplenia, the infection is more severe, but the hospital mortality rate is similar [10]. In our study, it has been observed that non-splenectomy in patients with asplenia was significantly associated with IPD, age, age group, LOHS, COPD, rheumatoid disease, AIDS, drug abuse, and any disease. The IPD prevalence (%) of the non-splenectomized patients by age group in Spain was the highest in those over 60 years age, and non-existent in those 0–4 years age for the splenectomized. These results constitute an advance in the knowledge of pneumococcal infection in patients with asplenia, deepening the differentiation regarding the intervention of the spleen. In addition to younger children and older people are at higher risk for IPD, functional or anatomical asplenia, chronic diseases, diabetes, asthma, smoking, alcoholism, congenital or acquired immunodeficiencies, and immunosuppression. In the Spanish 25-year period contemplated, there is evidence of epidemiological differences between non-splenectomized patients and IPD, including in clinical presentation, higher age-adjusted comorbidity index scores, and those of an older age.

In the current study, the incidence and fatality of IPD throughout the period 1997–2021 decreased. These numbers must be interpreted considering the effect of the COVID-19 pandemic, which may have caused fewer serious cases to be notified within the surveillance system. The incidence was higher in men than in women, in the group of 60 years of age or older, and in those under 4 years of age. The highest lethality was observed in patients over 60 years of age or older.

As indicated, asplenia is a risk factor for the development of severe bacterial infection. Hyposplenism is likely the most common presentation of this entity and has many etiologies [11]. The epidemiology of asplenia differs depending on its etiology. Nearly 100% of SCD patients will develop asplenia, and they are more prone to OPSI. Many other patients with similar hemoglobinopathies are at risk of splenomegaly and a possible need for splenectomy or may develop hyposplenism with the course of their disease (e.g., SCD) [12]. Individuals with splenic dysfunction, asplenia (functional or anatomical), or with complement deficiencies are at an increased risk of infection caused by bacteria such as SP (>50% of cases), *Haemophilus influenzae* type b, and *Neisseria meningitidis* [13]. The complication of greatest concern in patients with asplenia or hyposplenism is IPD or OPSI [14]. Patients with asplenia are also at risk for less common infections due to malaria, *Babesia*, and *Capnocytophaga* [15]. The most important pathogen is SP, which accounts for 40–60% of cases in splenectomized patients [5]. This could form the basis of new studies that complement what was described and increase knowledge about the risk of SP infection.

IPD is seen more frequently in the population older than 65 years and in those who present both immunosuppressive comorbidities (infection by human immunodeficiency virus, solid and hematological tumors, organ transplantation, functional asplenia or anatomical) and non-immunosuppressive comorbidities (cardiovascular or chronic respiratory disease, liver disease, alcoholism, smoking, cerebrospinal fluid fistulas, and cochlear implants) [15], an aspect that has been confirmed in the study and constituted risk groups [16]. In particular, this applies to comorbidities for patients with asplenia infected with SP.

Recommendations for pneumococcal vaccination in those undergoing splenectomy should be considered carefully, and if these findings are replicated, future vaccines should have broader coverage including additional serotypes affecting this population [10]. The risk of life-threatening infections is high in young children with asplenia [15]. In the study population, this aspect is relevant for IPD in children with asplenia aged 0–4 years (OR = 2.15) and significant in those over 60 years (OR = 2.83).

Prophylactic measures are important to reduce mortality in patients with asplenia; principally, education, vaccinations, and antibiotic prophylaxis [17]. The most serious complication of asplenia is overwhelming infection, with mortality rates as high as 50% in the absence of prevention strategies such as antibiotic prophylaxis and vaccines. As was commented and demonstrated, the risk for infection in the study population is generally higher in adults, particularly aged ≥60 years. The age-dependent risk may reflect a combination of indications for splenectomy and the risk of exposure to pathogens such as SP [18]. In patients with asplenia, daily antibiotic prophylaxis is recommended for SCD, with a 50% to 63% reduction in pneumococcal infection [19]. Therefore, this prophylaxis is recommended, plus immunization for preventing infection. In fact, the Infectious Diseases Society of America has recommended pneumococcal vaccinations for patients with asplenia and SCD for the prevention of IPD [20]. However, more research regarding the duration of antibiotic therapy is needed, given the epidemiologic shifts in colonization and infection due to newer vaccines [18]. Global coverage of the pneumococcal vaccine (PCV), which significantly reduces pneumonia, another of the main causes of death in children, does not reach 50% [21]. In 2012, the ACIP advised that high-risk adults (19–64 years) with functional or anatomic asplenia, immunocompromising conditions, cochlear implant, or cerebrospinal fluid leak, receive both PCV13 and PPSV23 in series and that high-risk adults with chronic conditions receive PPSV23 [22]. Additionally, antibiotic prophylaxis in children with asplenia is recommended but not always efficient [23]. In the immunocompromised population, vaccination is recommended in most countries, including in Spain. The great variability in European pneumococcal vaccination guidelines warrants European unification of the guidelines for better control of pneumococcal disease [24]. These findings could suggest the need for patients with asplenia to receive appropriate pneumococcal vaccinations.

The choice of antibiotic must be guided by the results of the susceptibility tests carried out on the pneumococcal isolated strains. There is no antimicrobial drug that includes its use for disease prophylaxis in the data sheet. Amoxicillin should be administered as the first-choice antibiotic and azithromycin or rifampicin as second-line antibiotics [9]. It should be noted that SP infection in patients with asplenia could be fast and overwhelming. For this, the prevention of infection is essential. Non-splenectomized patients should be vaccinated with the PCV and, in general, all patients with asplenia should consider vaccination, the use of prophylactic antibiotics, and knowing when to seek medical care [5].

The limitations of this study may refer to it being retrospective with inherently associated bias. Others could be related to missing data, associated errors in data abstraction, and managed data care in patient populations. Therefore, the results are only directly applicable to patients in managed hospital settings. Other limitations could be related to the readmission of patients with asplenia with dysfunction who undergo surgery, the lack of follow-up of patients, risk factors not included in CMBD, and other classifications of non-splenectomized not contemplated.

The key strengths of the current study are that only patients who truly did have diagnosed asplenia were included, with a huge sample size and observation time, and from a very wide geographic distribution. Although all patients had asplenia diagnoses, this covered a range of conditions, including total and partial splenectomy, various severe spleen injuries, and congenital problems. In conclusion, the study of patients with asplenia under its different modalities provides relevant information about the state of SP infection. This epidemiological tracking can serve, among other benefits, to better understand the comorbidities that affect them, the risk factors for the disease, the prediction of antibiotic use, and vaccination in public health. In addition to the surveillance system, this plays a fundamental role in the evaluation of the impact of vaccination on the incidence and lethality of the IPD, and on the effect that changes in the prevalence of circulating serotypes may have on the effectiveness of the vaccine, and on the appearance of cases due to resistant serotypes. This epidemiological information is necessary to define the most appropriate vaccination strategies. Identifying patients with asplenia (e.g., with functional hyposplenism) is important because simple measures such as vaccination against common infective microorganisms (e.g., *S. pneumoniae*, *N. meningitidis,* and *H. influenzae*) and antibiotic therapy when needed are considered beneficial in diminishing the frequency and gravity of the infections accompanying the syndrome [25].

## Figures and Tables

**Table 1:** Characteristics of the population.

Total		Total N = 132,257		Non-Infection N = 131,785		Infection N = 472		*p*-Value
Age, mean, SD		53.81 ± 21.99		53.79 ± 21.99		58.3 ± 20.03		0.000
Age group	0–4 years	1913	1.45%	1907	1.45%	6	1.27%	0.000
	5–19 years	10,476	7.92%	10,463	7.94%	13	2.75%	
	20–39 years	22,646	17.12%	22,574	17.13%	72	15.25%	
	40–59 years	34,696	26.23%	34,568	26.23%	128	27.12%	
	Over 60 years	62,526	47.28%	62,273	47.25%	253	53.60%	
Sex	Male	81,191	61.39%	80,876	61.37%	315	66.74%	0.017
	Female	51,066	38.61%	50,909	38.63%	157	33.26%	
Splenectomy	Yes	68,256	51.61%	68,079	51.66%	177	37.50%	0.000
	No	64,001	48.39%	63,706	48.34%	295	62.50%	
LOHS (Length of Hospital Stay), median, IQR		11 ± 16		11 ± 16		18 ± 22		0.000
AMI (Acute Myocardial Infarction)		2632	1.99%	2619	1.99%	13	2.75%	0.234
CHF (Congestive Heart Failure)		5909	4.47%	5875	4.46%	34	7.20%	0.004
PVD (Peripheral Vascular Disease)		5925	4.48%	5904	4.48%	21	4.45%	0.974
CeVD (Cerebrovascular Disease)		4066	3.07%	4042	3.07%	24	5.08%	0.011
Dementia		838	0.63%	832	0.63%	6	1.27%	0.080
COPD (Chronic Obstructive Pulmonary Disease)		11,938	9.03%	11,858	9.00%	80	16.95%	0.000
Rheumatoid Disease		1687	1.28%	1671	1.27%	16	3.39%	0.000
PUD (Peptic Ulcer Disease)		2465	1.86%	2454	1.86%	11	2.33%	0.453
Diabetes		14,730	11.14%	14,680	11.14%	50	10.59%	0.707
Diabetes and Complications		1298	0.98%	1293	0.98%	5	1.06%	0.863
HP/PAPL (Hemiplegia or Paraplegia)		1018	0.77%	1015	0.77%	3	0.64%	0.738
RD (Renal Disease)		5721	4.33%	5691	4.32%	30	6.36%	0.030
Cancer		25,214	19.06%	25,127	19.07%	87	18.43%	0.726
AIDS		1232	0.93%	1206	0.92%	26	5.51%	0.000
Pulmonary Circulation Disorders		3327	2.52%	3309	2.51%	18	3.81%	0.071
Hypertension, Complicated		5322	4.02%	5299	4.02%	23	4.87%	0.347
Paralysis		1018	0.77%	1015	0.77%	3	0.64%	0.738
Other Neurological Disorders		3333	2.52%	3309	2.51%	24	5.08%	0.000
Liver Disease		16,525	12.49%	16,461	12.49%	64	13.56%	0.483
Metastatic Cancer		17,308	13.09%	17,275	13.11%	33	6.99%	0.000
Obesity		4987	3.77%	4970	3.77%	17	3.60%	0.847
Alcohol Abuse		8876	6.71%	8828	6.70%	48	10.17%	0.003
Drug Abuse		2537	1.92%	2511	1.91%	26	5.51%	0.000
Any Disease		82,018	62.01%	81,677	61.98%	341	72.25%	0.000

**Table 2:** Characteristics of the population with splenectomy.

Splenectomy		Total N = 68,256		Non-Infection N = 68,079		Infection N = 177		*p*-Value
Age, mean, SD		54.78 ± 20.09		54.77 ± 20.1		57.23 ± 19.01		0.104
Age group	0–4 years	194	0.28%	194	0.28%	0	0.00%	0.475
	5–19 years	4586	6.72%	4579	6.73%	7	3.95%	
	20–39 years	11,300	16.56%	11,274	16.56%	26	14.69%	
	40–59 years	19,065	27.93%	19,011	27.92%	54	30.51%	
	Over 60 years	33,111	48.51%	33,021	48.50%	90	50.85%	
Sex	Male	40,520	59.36%	40,396	59.34%	124	70.06%	0.004
	Female	27,736	40.64%	27,683	40.66%	53	29.94%	
LOHS (Length of Hospital Stay), median, IQR		13 ± 18		13 ± 18		27 ± 27		0.000
AMI (Acute Myocardial Infarction)		985	1.44%	980	1.44%	5	2.82%	0.123
CHF (Congestive Heart Failure)		1645	2.41%	1633	2.40%	12	6.78%	0.000
PVD (Peripheral Vascular Disease)		1691	2.48%	1688	2.48%	3	1.69%	0.502
CeVD (Cerebrovascular Disease)		921	1.35%	915	1.34%	6	3.39%	0.018
Dementia		159	0.23%	159	0.23%	0	0.00%	0.520
COPD (Chronic Obstructive Pulmonary Disease)		4751	6.96%	4734	6.95%	17	9.60%	0.166
Rheumatoid Disease		645	0.94%	642	0.94%	3	1.69%	0.302
PUD (Peptic Ulcer Disease)		1456	2.13%	1450	2.13%	6	3.39%	0.247
Diabetes		6611	9.69%	6601	9.70%	10	5.65%	0.069
Diabetes and Complications		336	0.49%	335	0.49%	1	0.56%	0.890
HP/PAPL (Hemiplegia or Paraplegia)		228	0.33%	227	0.33%	1	0.56%	0.594
RD (Renal Disease)		1858	2.72%	1855	2.72%	3	1.69%	0.400
Cancer		18,305	26.82%	18,252	26.81%	53	29.94%	0.347
AIDS		424	0.62%	416	.61%	8	4.52%	0.000
Pulmonary Circulation Disorders		674	0.99%	672	0.99%	2	1.13%	0.848
Hypertension, Complicated		1471	2.16%	1467	2.15%	4	2.26%	0.923
Paralysis		228	0.33%	227	0.33%	1	0.56%	0.594
Other Neurological Disorders		1123	1.65%	1118	1.64%	5	2.82%	0.217
Liver Disease		4713	6.90%	4705	6.91%	8	4.52%	0.210
Metastatic Cancer		12,211	17.89%	12,188	17.90%	23	12.99%	0.089
Obesity		2225	3.26%	2218	3.26%	7	3.95%	0.602
Alcohol Abuse		3188	4.67%	3174	4.66%	14	7.91%	0.041
Drug Abuse		1029	1.51%	1025	1.51%	4	2.26%	0.411
Any Disease		43,105	63.15%	42,985	63.14%	120	67.80%	0.200

**Table 3:** Characteristics of the population with no splenectomy.

No Splenectomy		Total N = 64,001		Non-Infection N = 63,706		Infection N = 295		*p*-Value
Age, mean, SD		52.77 ± 23.8		52.74 ± 23.81		58.94 ± 20.63		0.000
Age group	0–4 years	1719	2.69%	1713	2.69%	6	2.03%	0.000
	5–19 years	5890	9.20%	5884	9.24%	6	2.03%	
	20–39 years	11,346	17.73%	11,300	17.74%	46	15.59%	
	40–59 years	15,631	24.42%	15,557	24.42%	74	25.08%	
	Over 60 years	29,415	45.96%	29,252	45.92%	163	55.25%	
Sex	Male	40,671	63.55%	40,480	63.54%	191	64.75%	0.668
	Female	23,330	36.45%	23,226	36.46%	104	35.25%	
LOHS (Length of Hospital Stay), median, IQR		10 ± 12		10 ± 12		13 ± 14		0.000
AMI (Acute Myocardial Infarction)		1647	2.57%	1639	2.57%	8	2.71%	0.880
CHF (Congestive Heart Failure)		4264	6.66%	4242	6.66%	22	7.46%	0.583
PVD (Peripheral Vascular Disease)		4234	6.62%	4216	6.62%	18	6.10%	0.722
CeVD (Cerebrovascular Disease)		3145	4.91%	3127	4.91%	18	6.10%	0.344
Dementia		679	1.06%	673	1.06%	6	2.03%	0.102
COPD (Chronic Obstructive Pulmonary Disease)		7187	11.23%	7124	11.18%	63	21.36%	0.000
Rheumatoid Disease		1042	1.63%	1029	1.62%	13	4.41%	0.000
PUD (Peptic Ulcer Disease)		1009	1.58%	1004	1.58%	5	1.69%	0.870
Diabetes		8119	12.69%	8079	12.68%	40	13.56%	0.651
Diabetes and Complications		962	1.50%	958	1.50%	4	1.36%	0.835
HP/PAPL (Hemiplegia or Paraplegia)		790	1.23%	788	1.24%	2	0.68%	0.386
RD (Renal Disease)		3863	6.04%	3836	6.02%	27	9.15%	0.024
Cancer		6909	10.80%	6875	10.79%	34	11.53%	0.685
AIDS		808	1.26%	790	1.24%	18	6.10%	0.000
Pulmonary Circulation Disorders		2653	4.15%	2637	4.14%	16	5.42%	0.270
Hypertension, Complicated		3851	6.02%	3832	6.02%	19	6.44%	0.759
Paralysis		790	1.23%	788	1.24%	2	0.68%	0.386
Other Neurological Disorders		2210	3.45%	2191	3.44%	19	6.44%	0.005
Liver Disease		11,812	18.46%	11,756	18.45%	56	18.98%	0.815
Metastatic Cancer		5097	7.96%	5087	7.99%	10	3.39%	0.004
Obesity		2762	4.32%	2752	4.32%	10	3.39%	0.433
Alcohol Abuse		5688	8.89%	5654	8.88%	34	11.53%	0.110
Drug Abuse		1508	2.36%	1486	2.33%	22	7.46%	0.000
Any Disease		38,913	60.80%	38,692	60.74%	221	74.92%	0.000

**Table 4:** Risk factors for IPD in patients with or without splenectomy.

Variable		Total		Splenectomy		No Splenectomy	
		OR (95% CI)	*p*-Value	OR(95% CI)	*p*-Value	OR (95% CI)	*p*-Value
Age	5–19 years	1(reference)	-	1 (reference)	-	1 (reference)	-
	0–4 years	2.15 (0.82–5.68)	0.121	-	-	3.41 (1.1–10.61)	0.034
	20–39 years	2.51 (1.39–4.54)	0.002	1.49 (0.64–3.44)	0.354	3.65 (1.56–8.58)	0.003
	40–59 years	2.71 (1.51–4.87)	0.001	1.83 (0.81–4.13)	0.145	3.65 (1.56–8.52)	0.003
	Over 60 years	2.83 (1.58–5.06)	0.000	1.74 (0.77–3.92)	0.182	4 (1.73–9.27)	0.001
Sex	Female	1 (reference)	-	1 (reference)	-	1 (reference)	-
	Male	1.27 (1.04–1.54)	0.016	1.63 (1.18–2.25)	0.003	1.09 (0.86–1.39)	0.465
Comorbidities	No	1 (reference)	-	1 (reference)	-	1 (reference)	-
	Yes	1.38 (1.1–1.73)	0.005	1.1 (0.77–1.57)	0.593	1.61 (1.2–2.16)	0.001
Causes of asplenia	Splenectomy	1 (reference)	-	-			
	No splenectomy	1.82 (1.51–2.19)	0.000				

Abbreviations: OR odds ratio, CI: confidence interval.

## Data Availability

The datasets analyzed in the current study are publicly available in the Hospital Discharge Records in the Spanish National Health System (CMBD) repository at https://www.mscbs.gob.es/en/estadEstudios/estadisticas/cmbdhome.htm (accessed on 13 July 2023). The information contained in this repository can be accessed without the need for any administrative permissions.

## Appendix A

### Demographic and Hospitalization Data

The Spanish CMBD contains routine hospital data including the diagnosis of spleen abnormalities, acquired absence of spleen, asplenia (congenital), and hyposplenism. For each CMBD discharge record, we considered asplenia diagnoses coded on the basis of the International Classification of Diseases (ICD), 9th Revision, ICD-9-MC (splenectomy, 41.5, 41.43; no splenectomy, 865, 759.0, 289.59) and 10th Revision, ICD-10-ES (splenectomy, 07TP0ZZ; no splenectomy, Q89.01, D73.3, D73.4, D73.5, D73.89, Q89.09). The definition of SP used according to the ICD-9-MC and ICD-10-ES codes was 481 and J13, respectively. To analyze the characteristics of the study population in patients with asplenia, all codes defining IPD were considered. For each case, specific data were gathered on age, sex, causes of asplenia, comorbidities, and risk factors for asplenia. In rates, the total number of total and perinatal mortality in Spain were also considered through the Spanish Instituto Nacional de Estadística (INE). To associate the precise hospitalization date for the population at risk in any given month, we assumed a constant birth rate throughout the year for the population denominators by months of age. The datasets generated and/or analyzed during the current study are available in the hospital discharge records in the Spanish CMBD repository.
